# Familial Parkinson's Disease/Parkinsonism

**DOI:** 10.1155/2015/736915

**Published:** 2015-04-19

**Authors:** Hiroyuki Tomiyama, Suzanne Lesage, Eng-King Tan, Beom S. Jeon

**Affiliations:** ^1^Department of Neurology, Juntendo University School of Medicine, Tokyo, Japan; ^2^Sorbonne Universités, UPMC Université Paris 6 UMRS 1127, Inserm U 1127, CNRS UMR 7225, Institut du Cerveau et de la Moelle Épinière (ICM), Paris, France; ^3^Department of Neurology, Singapore General Hospital and Neuroscience & Behavioral Disorders Program, Duke-NUS Graduate Medical School, National Neuroscience Institute, Singapore; ^4^Department of Neurology, Seoul National University Hospital, Seoul, Republic of Korea

Parkinson's disease (PD) is the second most common neurodegenerative disorder characterized by parkinsonism (bradykinesia, resting tremor, rigidity, and postural instability) with good response to L-dopa. Although the majority of PD patients are sporadic, it is now clear that genetic factors contribute to the pathogenesis of PD. Indeed,* PARK* 1-20 loci have been identified in typical and atypical parkinsonism. Furthermore, a new causative gene for PD was identified in Japanese families very recently. Knowledge and understanding of these conditions have led to the development of animal models, successful therapies, and novel tools to characterize these clinical conditions and provide better care to patients.

In this special issue, we can see 8 papers (original research articles and review articles) as follows.

H. Park et al. reviewed the epidemiologic, clinical, genetic, and pathologic features of parkinsonism in spinocerebellar ataxia (SCAs). They highlighted parkinsonism related to SCA2, SCA3, and SCA17 in Asia, especially in Korea. They showed that parkinsonism in SCAs has the geographic differences in prevalence. Further insights into parkinsonism in SCAs might give us the new concept in the pathologic mechanisms.

About* SNCA (alpha-synuclein, PARK1)*, we can see two papers. Y. Huang et al. investigated independent and joint effects of* MAPT and SNCA* on PD onset age. In their original article, they reported that the* SNCA* variants independently influence onset age of Parkinson's disease in Chinese and Australians. Then, M. A. Busquets et al. reviewed a hot button issue of the ability of alpha-synuclein to misfold in amyloid conformations and to spread via neuron-to-neuron transmission, suggesting a prion-like behavior. They described that the high neuronal toxicity of both mature fibres and oligomeric species, as well as the intracellular localization of the protein and the difficulty to be secreted, could be key factors impeding the prion ability of alpha-synuclein aggregates. These two papers had important discussions on the role of the* SNCA* gene and alpha-synuclein as the key molecule in PD/parkinsonism.

V. Drouet et al. reviewed the identified gene,* SYNJ1* (encoding for Synaptojanin 1), mutation in PD and discussed further insight into the neuropathological mechanisms. Recently, homozygous* SYNJ1* Arg258Gln mutation in one of* SYNJ1* functional domains was found in three unrelated families with early-onset atypical parkinsonism with bradykinesia, dystonia, and variable atypical symptoms such as cognitive decline, seizures, and eyelid apraxia.* SYNJ1* was designated as PARK20 most recently. Identification of* SYNJ1* can further support the fact that most of the known PD genes code for proteins playing a role in synaptic vesicle recycling and lipid metabolism, pointing out that synaptic maintenance is a key player in PD pathological mechanisms.

X. Yang et al. reviewed current knowledge about the* ATP13A2* gene, clinical characteristics of patients with PD-associated* ATP13A2* mutations, and models of how the ATP13A2 protein may help prevent neurodegeneration by inhibiting *α*-synuclein aggregation and supporting normal lysosomal and mitochondrial function. They also discussed another* ATP13A2* mutation that was associated with neuronal ceroid lipofuscinoses (NCLs) and they proposed a single pathway whereby* ATP13A2* mutations may contribute to NCLs and parkinsonism.

S. Scuderi et al. discussed that alternative splicing in* PARK2* generates the expression of different PARK2 (Parkin) protein isoforms and leads to selective degeneration of dopaminergic neurons based on evidences in human, rat, and mouse brains. Finally, they described that understanding* PARK2* alternative splicing could open up new scenarios for the resolution of some parkinsonian syndrome. Also, A. Kh. Alieva et al. reported involvement of endocytosis and alternative splicing in the formation of the pathological process in the early stages of PD. They demonstrated a significant change in the levels of transcripts included in the large groups of processes associated with the functioning of the immune system and cellular transport. Moreover, a significant change in the splicing of genes involved in cellular-transport processes was shown in their study. Alternative splicing should be considered as another pathway of regulation of the gene expression which can lead to neurodegeneration.

A. A. Gopalai et al. conducted a large genetic study and reported common* LRRK2 (PARK8)* G2385R and R1628P variants associated with an increased risk of PD in the Malaysian population. They provided new positive data on the* LRRK2* variants in Asian (Chinese, Taiwanese, Japanese, Singaporean, and Korean) populations.

In this special issue, they highlighted advances in genetic findings of PD/parkinsonism and findings about the disease mechanism and pathogenesis which can lead to therapeutic strategy for PD/parkinsonism. Their original studies and reviews will stimulate the continuing efforts to understand the molecular pathology underlying PD/parkinsonism, the development of strategies to treat these conditions, and the evaluation of outcomes.

